# Mitochondrial Dysfunction as an Underlying Cause of Skeletal Muscle Disorders

**DOI:** 10.3390/ijms232112926

**Published:** 2022-10-26

**Authors:** Tsung-Hsien Chen, Kok-Yean Koh, Kurt Ming-Chao Lin, Chu-Kuang Chou

**Affiliations:** 1 Department of Internal Medicine, Ditmanson Medical Foundation Chia-Yi Christian Hospital, Chiayi 60002, Taiwan; 2 Division of Gastroenterology and Hepatology, Department of Internal Medicine, Ditmanson Medical Foundation Chia-Yi Christian Hospital, Chiayi 60002, Taiwan; 3 Institute of Biomedical Engineering and Nanomedicine, National Health Research Institutes, Zhunan 35053, Taiwan; 4 Obesity Center, Ditmanson Medical Foundation Chia-Yi Christian Hospital, Chiayi 60002, Taiwan

**Keywords:** mitochondrial dysfunction, skeletal muscle disorders, OXPHOS, mtDNA mutation, mitochondrial dynamics, mitophagy, mitochondrial chaperone protein

## Abstract

Mitochondria are an important energy source in skeletal muscle. A main function of mitochondria is the generation of ATP for energy through oxidative phosphorylation (OXPHOS). Mitochondrial defects or abnormalities can lead to muscle disease or multisystem disease. Mitochondrial dysfunction can be caused by defective mitochondrial OXPHOS, mtDNA mutations, Ca^2+^ imbalances, mitochondrial-related proteins, mitochondrial chaperone proteins, and ultrastructural defects. In addition, an imbalance between mitochondrial fusion and fission, lysosomal dysfunction due to insufficient biosynthesis, and/or defects in mitophagy can result in mitochondrial damage. In this review, we explore the association between impaired mitochondrial function and skeletal muscle disorders. Furthermore, we emphasize the need for more research to determine the specific clinical benefits of mitochondrial therapy in the treatment of skeletal muscle disorders.

## 1. Introduction

Mitochondria are important energy factories in cells, and hundreds of mitochondria provide the required cellular energy. Mitochondrial diseases are caused by defects in the mitochondria and resulting symptoms. Mitochondrial disease may affect more than one cell type, tissue, or organ, resulting in multisystem disease [1]. Muscle disease is a common feature of mitochondrial disease due to the high energy demands of muscle cells. Mitochondria within muscle tissue consume approximately 85–90% of the oxygen in the cell to allow the mitochondrial metabolic pathway to function [2]. During energy demand, the coupling of upstream oxidative processes, such as glycolysis, β-oxidation, and the Krebs cycle with oxidative phosphorylation (OXPHOS), results in the release of adenosine triphosphate (ATP) [3]. The activity of mitochondrial metabolic enzymes and the OXPHOS complex, respiration, protein synthesis, and ATP production rates in skeletal muscle are functionally impaired with increasing age [4,5,6]. Skeletal muscle has high demands for ATP production from both glycolysis and OXPHOS. Thus it is also susceptible to the production of reactive oxygen species (ROS), including singlet oxygen, superoxide, and hydroxyl radicals [7]. Antioxidant mechanisms maintain free radical homeostasis and release ROS-scavenging enzymes, such as superoxide dismutase, catalase, and glutathione peroxidase, in normal physiology [8].

## 2. Mitochondria

Mitochondria are elongated, cylindrical, double-membrane-bound organelles located in the cytoplasm of eukaryotic cells that vary widely in size [9,10]. Mitochondria not only influence and often coordinate metabolism, utilizing aerobic respiration to generate most of the ATP through OXPHOS, but also participate in complex cellular signaling events, such as those involved in regulating calcium ion (Ca^2+^) homeostasis, cellular division, differentiation, senescence, apoptosis, and death events, as well as the maintenance of control over the cell cycle and cell growth [11,12,13,14]. The circular mitochondrial genome (mtDNA) encodes 13 catalytic proteins of the OXPHOS pathway, 22 mitochondrial transfer RNA (mt-tRNA), and 2 mitochondrial ribosomal RNA (mt-rRNA) [15]. The majority of OXPHOS complex subunits, assembly factors, and all proteins ensure mtDNA maintenance are nuclear encoded. The five multimeric OXPHOS complexes are embedded in the lipid bilayer of the inner mitochondrial membrane, including complex I to complex V. Phosphorylation of the OXPHOS machinery is responsible for OXPHOS-dependent functions (such as ROS generation) that regulate OXPHOS complex assembly and stability and fine-tune bioenergetic control and responses to cellular stimuli [16].

### 2.1. The Role of Mitochondria in Metabolism

**Figure 1:**
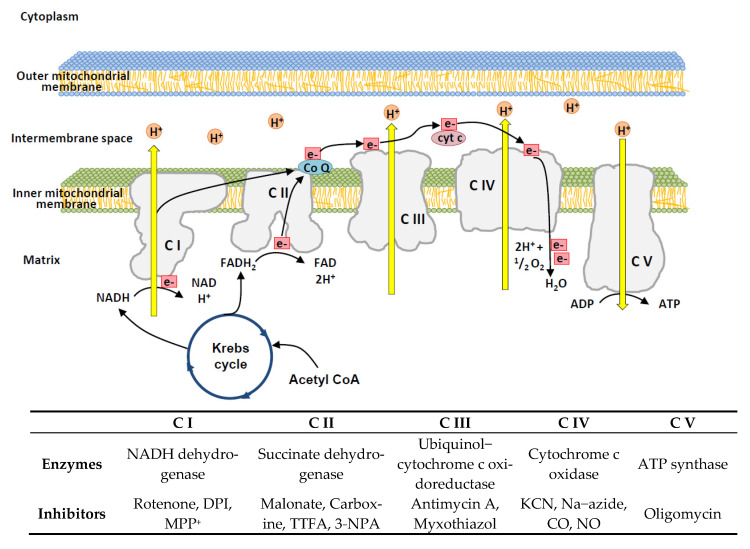
Schematic model of the oxidative phosphorylation (OXPHOS) process and relative inhibitors. In the electron transport chain, the reduced substrates NADH and FADH_2_ are oxidized by NADH−ubiquinone oxidoreductase and succinate−CoQ reductase, respectively. Finally, cytochrome c oxidase reduces molecular oxygen to water using electrons from reduced cytochrome c. The Krebs cycle is one of the main suppliers of redox equivalents. OXPHOS inhibitors are shown in the lower box. 3-NPA, 3-nitropropionic acid; ATP, adenosine triphosphate; C I, Complex I; C II, Complex II; C III, Complex III; C IV, Complex IV; C V, Complex V; CO, carbon monoxide; CYTC, cytochrome c; CoQ, coenzyme Q10; DPI, diphenyleneiodonium; KCN, potassium cyanide; MPP, 1-methyl-4-phenylpyridinium; Na−azide, sodium azide; NADH, nicotinamide adenine dinucleotide; NO, nitric oxide; TTFA, thenoyltrifluoroacetone.

Multiple biosynthetic and catabolic pathways occur within mitochondria. For example, mitochondria produce the ATP needed by cells through respiration and regulation of cellular metabolism. In each mitochondrion, ATP is produced using sugars, fatty acids, or amino acids combined with oxygen. Core reactions involved in ATP production include the citric acid cycle or the Krebs cycle and OXPHOS. Electrons are transported from nicotinamide adenine dinucleotide (NADH) to oxygen (O_2_) through OXPHOS complex I to complex IV (Figure 1). The Krebs cycle occurs in the matrix of mitochondria with the net result of utilizing carbohydrates from glycolysis to generate ATP, carbon dioxide (CO_2_), NADH, and flavin adenine dinucleotide (FADH) [17]. While mitochondria are important calcium storage organelles that regulate respiration according to metabolic demand, the large calcium buffer capacity of mitochondria also contributes to the maintenance of cytosolic calcium homeostasis. The disruption of calcium homeostasis can lead to cell death. Both apoptotic and necrotic cell death pathways can be initiated by events involving abrupt calcium release from mitochondria [18]. Mitochondrial Ca^2+^ uptake plays an important regulatory role in mitochondrial activity, metabolism, and energy production, and mitochondrial calcium level has an important role in regulating metabolic pathways, such as the Krebs cycle. Mitochondria are intracellular calcium regulatory and storage organelles wherein calcium import is controlled by the mitochondrial calcium uniporter complex, which is composed of pore-forming proteins, such as mitochondrial calcium uniporter (MCU), mitochondrial calcium uniporter-dominant negative subunit beta (MCUβ), a single-pass membrane protein with aspartate rich tail 1 (SMDT1), and the regulatory proteins mitochondrial calcium uptake 1 (MICU1) and mitochondrial calcium uptake 2 (MICU2). After an increase in cytosolic Ca^2+^, the uptake of Ca^2+^ by mitochondria from the mitochondrial intermembrane space (IMS) also increases via steps involving the binding of Ca^2+^ ions to MICU proteins, which opens channels and allows Ca^2+^ ions to be transported into mitochondria. In the matrix, calcium ions are shown to activate the Krebs cycle, stimulate the activity of the respiratory chain, and increase ATP synthesis [19,20].

### 2.2. Mitochondrial Dysfunction

Mitochondria are the most important sources of ROS in the eucaryotic cell, and these ROS are mainly produced by complexes I and III of the electron transport chain (ETC) due to the reaction of oxygen with electrons leaked from the respiratory chain [21]. To cope with excessive ROS, mitochondria are equipped with an antioxidant system involving enzymes, such as superoxide dismutase and glutathione peroxidase, that react with and scavenge ROS and serve as molecular chaperones that counteract ROS-induced errant protein folding [22,23]. Mitochondrial calcium homeostasis, respiration, ATP synthesis, and ROS production are an interwound network, and molecular defects in one component can have sequential effects on many other components and result in deleterious outcomes beyond the mitochondria, including in the cytoplasm and nucleus. For example, increased intramitochondrial Ca^2+^ can increase pyruvate dehydrogenase activity and ATP synthesis. Mitochondrial defects are manifested in decreased ATP production [24], reduced respiration [25], and increased ROS production [26,27].

Mitochondrial number, size, and density, as well as mitochondrial DNA and protein expression, decrease with age [28,29]. The level of mitochondrial mutations also increases with age [30]. Mitochondrial mutations and subsequent dysfunction that directly affects cellular metabolism can lead to disease. For example, major mutations that have been identified in mtDNA include point mutations in protein-coding regions and mt-tRNA genes that alter mitochondrial protein synthesis, as well as large-scale mtDNA deletions [31]. The pathogenicity of mtDNA mutations is due to heterogeneity and occurs when the proportion of mutant mtDNA exceeds a threshold effect for a given limit. In addition, circulating mtDNA gradually increases with age and correlates with serum inflammatory markers [32].

### 2.3. Mitochondrial Dynamics

**Figure 2:**
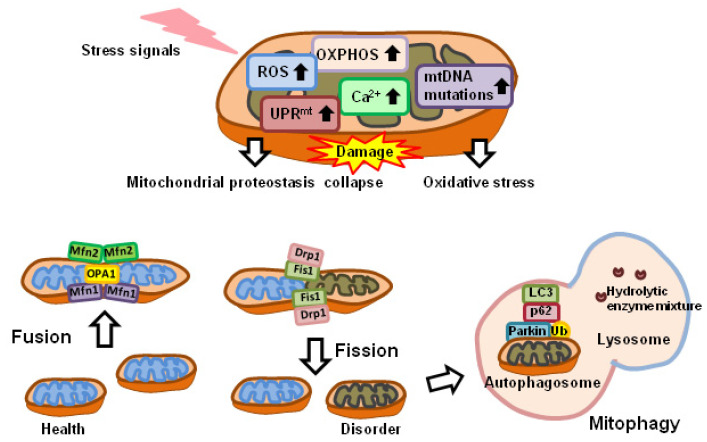
Mitochondrial dysfunction, dynamics, and mitophagy. Mitochondrial dysfunction is accompanied by increased ROS production, UPR^mt^, Ca^2+^, and mtDNA mutations, as well as altered cellular respiration and altered metabolism. Mitochondrial fission is the splitting of mitochondria into two smaller mitochondria. DRP1 is recruited by FIS1 anchored on the outer mitochondrial membrane. Mitochondrial fusion refers to the merging of two mitochondria into one. MFN1 and MFN2 mediate mitochondrial outer membrane fusion, and OPA1 mediates mitochondrial inner membrane fusion. After mitochondrial depolarization, PINK1 accumulates on the outer mitochondrial membrane (OMM) and recruits Parkin to the mitochondrial surface. Parkin ubiquitinates various OMM proteins. Polyubiquitinated proteins can be recognized by adaptor molecules, such as p62. These proteins interact with lipidated LC3 through the LC3-interacting region (LIR) motif, promoting the autophagosome encapsulation of damaged mitochondria. LC3, light chain 3; MFN1, mitofusin1; MFN2, mitofusin2; mtDNA, mitochondrial DNA; OPA1, optic neurotrophin 1; OXPHOS, oxidative phosphorylation; p62, p62/Sequestosome 1; ROS, reactive oxygen species; Ub, ubiquitin; UPR^mt^ mitochondrial unfolded protein response.

Mitochondrial dynamics involve organelle fusion and fission, as well as ultrastructural remodeling of membranes [14], and are important for the removal of aging and damaged mitochondria [33]. Mitochondrial dynamics refer to the morphological changes of mitochondria, including size and shape, as well as intracellular distribution, and are closely coordinated with cellular biological and metabolic processes. The alteration of mitochondrial dynamics can result from excessive ROS [34], perturbation of Ca^2+^ homeostasis [35], and as part of the processes of autophagy [36], mitophagy [37], and apoptosis [38]. Cellular bioenergetics can be regulated by changes in mitochondrial dynamics (Figure 2) [39,40]. The inhibition of mitochondrial fusion-essential protein mitofusin-2 (MFN2) increases ROS production and the expression of mitogen-activated protein kinase 8 (MAPK8) and nuclear factor kappa B (NFκB) in activated B-cell signaling, resulting in decreased insulin signaling and glucose uptake [41]. The mitochondrial inner membrane (MIM) fusion protein OPA1, mitochondrial dynamin-like GTPase (OPA1), and fission, mitochondrial 1 (FIS1) are known to be elevated in athletes [42]. In addition, *MFN2* messenger RNA (mRNA) levels and insulin expression are increased in muscle cells after bariatric surgery, thereby altering the efficiency of glucose oxidation [43]. However, excessive mitochondrial fission is also associated with metabolic dysfunction in skeletal muscle. Abnormal expression of dystrophin-related protein 1 (DRP1), which is responsible for mitochondrial fission, leads to ceramide-induced hydrogen peroxide (H_2_O_2_) production and impaired mitochondrial bioenergetics [44].

### 2.4. Mitochondrial Control of Stress Responses

Autophagy is a major intracellular degradation system with degradative capacity via lysosomes. Mitochondria play a key role in autophagy as a substrate for degradation (i.e., mitophagy) [45] and are specifically protected by autophagic machinery, ensuring energy production during stress responses [46,47]. The relationship between mitochondria and autophagy-centered stress responses is twofold. Damaged but intact mitochondria can be removed by mitochondrial autophagy (Figure 2). Autophagy, under normal conditions, maintains cell renewal and the repair of damaged cells [48] and is also involved in cellular development and differentiation [49]. However, cells can cope with hypoxia, nutrient deprivation, and various stress conditions by increasing autophagy [50].

Mitochondrial ROS appear to be required for autophagy [51], and hypoxia-induced mitochondrial autophagy, in particular, is mediated by protein kinase AMP-activated catalytic subunit alpha 2 (PRKAα2) [52,53,54]. Mitochondrial ROS protect against excessive immune responses by macrophages and extend the lifespan through mitotic mechanisms [55]. During normal aging, mitophagy levels markedly decrease [56,57]. When mitophagy decreases, it may exacerbate the vicious cycle of abnormal oxidative stress-induced age-related tissue damage [58].

### 2.5. Regulation of Apoptosis and Cell Death by Mitochondria

Permeabilization of the mitochondria outer membrane (MOM) is critical for apoptosis and regulated necrosis [59,60,61]. The voltage-dependent anion channel (VDAC) of the MOM, e.g., VDAC1 and VDAC2, facilitate ion fluxes between mitochondria and cytosol and regulate important cell functions via regulating adenosine diphosphate (ADP) and ATP metabolites pools. Dysfunction at VDACs can lead to catastrophic events, such as cell death [62]. The integrity of mitochondria is integral to cell fate. Mitochondria are guarded by a double membrane consisting of MOM and MIM, and the permeabilization of these membranes (MOMP and MIMP), which denotes the transitional process of releasing mitochondrial contents into the cytosol, is regulated by pore-forming proteins associated with the MOM, such as members of the BCL2 apoptosis regulator (BCL-2) family, BCL2 associated X, apoptosis regulator (BAX), and BCL2 antagonist/killer (BAK) [63]. MOMP resulting from BAX/BAK-associated pores leads to the release of apoptotic factors, such as cytochrome C, somatic (CYTC) and apoptotic peptidase-activating factor 1 (APAF1), from the intramembrane space and induces cell apoptosis. MIMP and the further widening of BAX/BAK pore result in the release of mtDNA, which is a potent trigger of innate immune response and inflammation. Thus, the induction of MOMP can result in a pro-inflammatory outcome, in addition to activating caspases and apoptotic cell death [64]. Following the DNA damage events, the release of apoptosis-inducing factors in response to IMS leads to the activation of DNA damage-associated enzyme polymerization (ADP-ribose polymerase, PARP) [65,66,67] by a cascade of events involving apoptosomes that are initiated by caspase-9 activation to cleave downstream caspase-3, -6, and -7 [68]. Abnormal level of ROS/reactive nitrogen species (RNS) level, death receptor activation by its ligands, DNA damage, Ca^2+^ dysregulation, e.g., cytosolic Ca^2+^ overload, endoplasmic reticulum (ER) stress, alterations in BCL-2 family protein expression, and other disturbances have been shown to be capable of triggering apoptosis [60,69].

## 3. Mitochondrial Dysfunction Affecting Skeletal Muscle

Disorders associated with skeletal muscle abnormalities resulting from mitochondrial dysfunction include mitochondrial myopathy, Duchenne muscular dystrophy, cachexia, and sarcopenia. Muscle fatigue, weakness, and exercise intolerance are the main symptoms of mitochondrial myopathy. Muscular dystrophy is characterized by progressive muscle weakness and wasting and histopathologically characterized by diffuse changes in fiber size, necrosis, fiber regeneration, fibrosis, atrophy, and, sometimes, inflammatory responses [70]. Cachexia is characterized by muscle and fat wasting leading to progressive weight loss [71]. In sarcopenia, the number of muscle fibers is reduced, resulting in decreased cross-sectional area and defective regeneration. Sarcopenia is a syndrome clinically defined by the loss of muscle mass and strength, which is multifactorial and particularly common in aging individuals. Cachexia is also a clinically defined syndrome that involves, in addition to severe sarcopenia, the features of systemic inflammation and metabolic dysfunction, such as muscle wasting. Mitochondrial dysfunction is the cellular and molecular basis underlying almost every feature of sarcopenia and cachexia [72]. In addition, the pathology of Huntington’s disease primarily manifests in the brain and consists of neuronal degeneration with mitochondrial defects occurring in the affected neurons. The skeletal muscles of patients with Huntington’s disease also show abnormal function via both neuronal degeneration and mitochondrial dysfunction [73].

### 3.1. Effects of Stress on Skeletal Muscle

**Figure 3:**
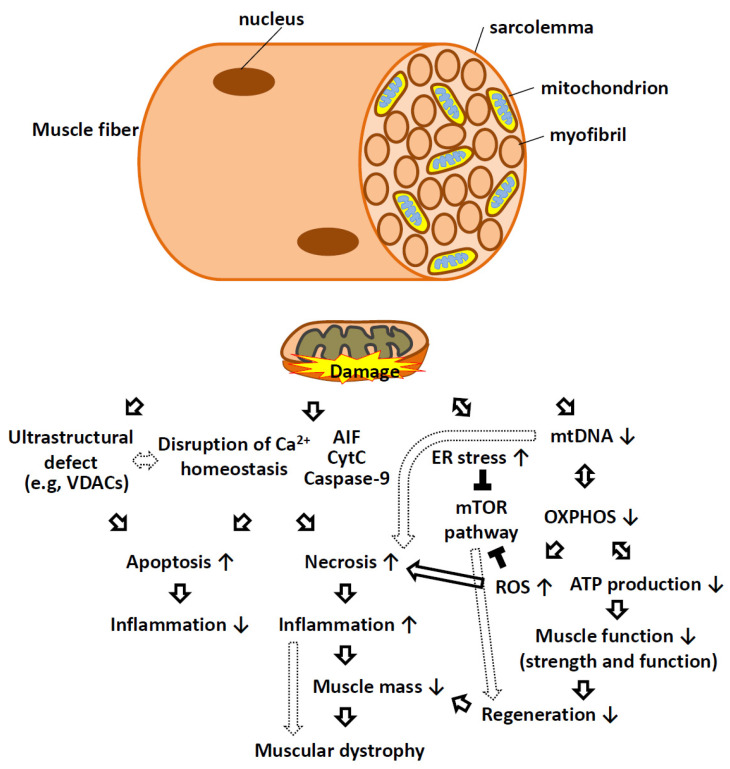
Schematic representation of mitochondrial dysfunction as causative factors in muscular dystrophy. In skeletal muscle fibers, mitochondria are located between myofibrils, surrounding the nucleus, and at high density within the sarcolemma. Mitochondrial dysfunction can manifest through loss of structural integrity during MOMP and MIMP and result in the release of CYTC, AIF, caspase-9, and calcium into the cytosol, triggering apoptotic and necrotic cell death programs. Ultrastructural defects, insufficient or defective mtDNA replication and aberrant protein expression can lead to impaired oxidative phosphorylation, insufficient respiration, and reduced ATP synthesis. Defects in respiration often result in elevated ROS production that can have consequences beyond the mitochondria. Mitochondrial dysfunction often connects with ER stress in that both can activate various signaling pathways (e.g., MTOR) that interfere with the normal trophic signaling necessary for muscle cell growth and proliferation. Reduced cell replication, increased cell death, and reduced anabolic responses lead to reduced muscle mass in patients with muscular dystrophy. AIF, apoptosis-inducing factor; ATP, adenosine triphosphate; ER, endoplasmic reticulum; mtDNA, mitochondrial DNA; MTOR, mammalian target of rapamycin kinase; OXPHOS, oxidative phosphorylation; ROS, reactive oxygen species; VDAC, voltage-dependent anion channel. Solid arrows indicate direct effects, while the dotted arrow indicates indirect effects. The ↑ bar indicates an increase, while the ↓ bar indicates a decrease. The black T bar indicates an inhibitory effect.

Mitochondrial damage leads to myopathy through a variety of factors, including mtDNA deletion mutations [74], Ca^2+^ signaling [75], loss of OXPHOS, defects in mitochondrial dynamics [76], abnormal mitochondrial structure and morphology [77], altered energy metabolism, mitophagy, and apoptosis. Muscular dystrophy can also be caused by mitochondrial mutation or dysfunction (Figure 3).

#### 3.1.1. Reactive Oxygen Species

The increase in ROS observed in cases of severe muscle disuse [78], sarcopenia [79], and muscular dystrophy [80] is associated with fiber atrophy and necrosis. Abnormal oxidative stress is regulated by many transcription factors, including NFκB [81], tumor protein p53 (TP53) [82], and hypoxia-inducible factor 1 subunit alpha (HIF1α) [83], resulting in the increased expression of growth factors, inflammatory cytokines, chemokines, and cell cycle regulators. Furthermore, abnormal oxidative stress inhibits the protein kinase B family (AKT)/mechanistic target of the rapamycin kinase (MTOR) pathway, which regulates the cell cycle and subsequently inhibits protein synthesis, thereby leading to muscle atrophy [84].

#### 3.1.2. Ca^2+^ Imbalance

The interruption of Ca^2+^ signaling leads to extensive cellular dysfunction and is associated with many muscle diseases, including myopathy, hypoxia, cachexia, sarcopenia, and heart failure. For example, calpain, which is activated by elevated intracellular Ca^2+^, is the best-characterized calcium-dependent protease. Following an increase in Ca^2+^ concentration, excessive calpain activation can break down a wide array of substrates, including cytoskeleton and ion channels. The breakdown of sodium channels would increase sodium concentration and result in the further overloading of calcium. The activation of calpain leads to skeletal muscle atrophy [85]. When muscle cells are stimulated, cytosolic Ca^2+^ increases, thereby increasing the mitochondrial uptake of Ca^2+^ [86]. Before muscle degeneration, muscle fibers become structurally unstable and highly permeable to the extracellular environment, leading to excessive Ca^2+^ influx that results in protease/lipase activation and intramitochondrial Ca^2+^ overload, which ultimately disrupts cellular Ca^2+^ homeostasis and leads to cell death [87]. Mitochondrial swelling leads to dysfunction that results in the inappropriate opening of the mitochondrial permeability transition pore (mPTP) and leads to the apoptosis and necrosis of muscle cells [88]. In addition, reduced MCU results in a reduced ability of skeletal muscle mitochondria to accumulate Ca^2+^ under stimulated and resting conditions, resulting in decreased muscle fiber size and muscle atrophy [89]. In skeletal muscle fibers silenced by the *MCU* gene, cellular basal and maximal respiration are reduced, and reduced OXPHOS leads to cellular metabolism and upregulation of fatty acid pathways [90].

#### 3.1.3. Aberrant Mitochondrial Chaperone Function

The mitochondrial unfolded protein response (UPR^mt^), caused by unfolded or misfolded proteins in mitochondria that are beyond the processing capacity of chaperone proteins, is a mitochondrial-related cellular stress response [91]. Heat shock protein 60 (HSP60) is a key chaperone in UPR^mt^ activation. HSP60 is an important mitochondrial stress protein that contributes to protein folding in the mitochondrial matrix space. HSP60 has also been shown to play a key role in increasing calpain activity in protein synthesis, folding, and delivery of misfolded proteins to proteolytic enzymes in the mitochondrial matrix [92]. UPR^mt^, a mitochondrial-to-nuclear signaling pathway regulated by the human homolog activating transcription factor 5 (ATF-5), is an important mechanism for repair in the event of mitochondrial dysfunction and a mediator of mitochondrial stress-induced autophagy [93]. UPR^mt^ also promotes an increase in glycolysis and amino acid catabolism genes while inhibiting the Krebs cycle and OXPHOS-encoding genes, relieving mitochondrial stress, and/or altering cellular metabolism to promote survival [94]. ER stress induces mitochondrial stress, and the HSP60 is also a regulator of the MTOR complex 1—sterol regulatory element-binding transcription factor 1 (SREBP1)—signaling that controls lipid metabolism [95].

HSP60 is not only an essential mitochondrial chaperone, but it also plays a key role outside the mitochondria. HSP60 is involved in the maintenance of skeletal muscle adaptation during exercise and skeletal muscle homeostasis in muscle pathology [92]. Overexpression of HSP60 in myoblasts induces PPARG coactivator 1 alpha (PPARGC1α) expression [96]. Mitochondrial homeostasis and OXPHOS in skeletal muscle are regulated by PPARGC1α, a master regulator of mitochondrial biosynthesis that is influenced by contractile activity. PPARGC1α activation leads to an increase in oxidative type I and IIa fibers and a decrease in the proportion of glycolytic type IIb fibers [97]. HSP60, when overexpressed in sarcopenia and cachexia, can improve muscle performance and reduce cachexia [92]. HSP60 can interact with the immune system to initiate or maintain inflammation, affecting the adaptation of skeletal muscle to exercise and tissue regeneration. In contrast, the overexpression of HSP60 has negative effects during development, as demonstrated by the developmental defects and excessive apoptosis observed after HSP60-induced heart failure in neonatal mice [98].

In addition, GrpE-like 1 (GRPEL1), a co-chaperone of mitochondrial HSP70 (mtHSP70), is an essential nucleotide exchange factor in mammalian mitochondria. The deletion of GRPEL1 induces a strong proteotoxic stress response in the cytoplasm and mitochondrial matrix, inhibits protein entry into mitochondria, and impairs the correct folding of mtHSP70. This results in the accumulation and aggregation of misfolded and mistargeted proteins. Thus, loss of GRPEL1 causes very rapid muscle atrophy and associated effects [99].

### 3.2. mtDNA Mutations and OXPHOS Dysfunction

Mutations in mtDNA or the nuclear genome (nDNA) induce mitochondrial myopathy [100]. Mutations in nDNA cause defects in OXPHOS, resulting in reduced activity of the respiratory chain enzyme complex. Mitochondrial oxidative damage is common in sarcopenia, resulting in ETC abnormalities and an increased incidence of mtDNA mutations [101]. Reduced respiratory chain activity in spinal muscular atrophy is associated with reduced mtDNA content [102]. In Duchenne muscular dystrophy, mitochondrial respiratory complexes I, III, and IV, as well as specific subunits of ATP synthase, are reduced, and the mitochondrial network is disturbed [103]. In mitochondria isolated from dystrophic skeletal muscle, maximal ATP synthesis was found to be drastically reduced [104]. Disruption of OXPHOS can also lead to mitochondrial myopathy [105]. During aging, satellite cell replication to replenish muscles is reduced, and the number of mitochondria per muscle cell also decreases. As a result, the OXPHOS of aging muscles is functionally insufficient, reminiscent of the mitochondrial myopathy observed in muscular dystrophy. Insufficient ATP production in aging muscle is compensated by enhanced cytoplasmic glycolysis, thereby increasing lactate and reducing pH, which leads to fatigue and reduced excitation-force coupling of muscle cells [106]. Deficiency in the number and function of mitochondria can lead to insufficient fatty acid oxidation and impair the regeneration of muscle cells [107]. Disruption in the oxidation of fatty acids makes greater efficiency of peroxisomes and mitochondria essential; without this, muscle regeneration further impaired [107]. Alterations in mitochondrial function also lead to increased ROS production and abnormal oxidative stress, further promoting mitochondrial damage [108]. In addition, decreased protein expression of CYTC oxidase and ATP synthase disrupts the oxidative capacity of skeletal muscle mitochondria in cachexia [109,110]. In muscular dystrophy, the level of the electron carrier ubiquinone (coenzyme Q10, CoQ10) in the ETC was found to be reduced.

### 3.3. Mitochondrial Dynamics and Mitophagy Imbalance

Mitochondria can reshape their morphological dynamics through mitochondrial fusion and fission in response to muscle signals [111]. Deletion of *MFN2* in skeletal muscle also reduces the adaptive control of autophagy and mitochondrial activation [112]. The knockout of *MFN1/2* in skeletal muscle resulted in more mtDNA mutations and tissue atrophy [113]. Likewise, cachexia patients showed altered levels of mitochondrial dynamic proteins MFN2 and FIS1 [114]. Additionally, deletion of the fission-related *DRP1* gene led to skeletal muscle atrophy and degeneration [35], whereas disruption of mitochondrial homeostasis in cachexia resulted in decreased mitochondrial function and related proteins [115,116,117], such as CYTC, succinate dehydrogenase complex flavoprotein subunit A (SDHA), OXPHOS complex IV, transcription factor A, mitochondrial (TFAM), translocase of outer mitochondrial membrane 20 (TOMM20), and mtDNA content [116,118,119,120].

Dystrophia myotonica 1 protein kinase A (DMPK-A) is localized in mitochondria and provides antioxidant and anti-apoptotic signals required for proper muscle fiber function and differentiation [121]. The binding and accumulation of DMPK-A in the MOM leads to changes in mitochondrial structure and morphology, ultimately inducing autophagy [122]. The expression levels of mitophagy-related proteins, such as microtubule-associated protein 1 light chain 3 beta (MAP1LC3β) [123], Parkin, PTEN induced kinase 1 (PINK1), and BCL2 interacting protein 3 (BNIP3), are altered in cachexia [115]. Mitophagy-related proteins such as PINK1 and Parkin are increased in cachectic skeletal muscle, activating mitochondrial degradation [116,124].

The UPR in the ER (UPR^er^) also affects gene expression related to protein degradation in atrophic muscles [125]. Uncontrolled protein aggregation due to the impairment or overburdening of the ubiquitin–proteasome and autophagy–lysosome systems leads to mitochondrial damage, aggregation-like residues, hyper-ubiquitinated proteins, and induction of autophagy, leading to muscle fibrotic injury [126]. Protein quality control is important for muscle cells, and skeletal muscle is not as resistant to protein stress as cardiomyocytes [127]. It is well known that Atrogin-1 and muscle-specific RING finger protein 1 (MURF1) are two ubiquitin E3 ligases of the ubiquitin–proteasome system that use eukaryotic translation initiation factor 3 subunit F (EIF3F) and myosin chain as their main substrates, respectively. Their expression in muscles is regulated by the forkhead box O (FOXO) transcription factor [128]. Decreased AKT activation in atrophic muscle promotes phosphorylation and translocation of FOXO to the nucleus, thereby promoting proteolysis by increasing the expression of Atrogin-1 and MURF1 [125,129]. Sarcopenia is due to decreased protein synthesis [130] and dysregulation of the proteasomal degradation pathway [131], resulting in intramuscular fat accumulation [132] and age-related lower satellite cell content and function [133]. In sarcopenia and skeletal muscle aging, mitophagy is increased, but the lysosomal function is impaired, suggesting that lysosomal dysfunction may lead to the accumulation of damaged mitochondria [134]. An imbalance between protein degradation and protein synthesis is another main cause of skeletal muscle loss in cachexia [135].

### 3.4. Ultrastructural Defects and Dysregulation

Muscle atrophy is characterized by wasting or loss of muscle mass, accompanied by reductions in the muscle fiber cross-sectional area, muscle volume, and muscle protein content [136]. Ultrastructural defects in skeletal muscle mitochondria and increased apoptosis are common in Ullrich congenital muscular dystrophy [88,137,138]. Muscle cell ultrastructure in Bethlem myopathy and Ullrich congenital muscular dystrophy show mitochondrial swelling, matrix hypodensity, disorganized cristae, heterotypic sacs associated with dilated sarcoplasmic reticulum, and altered-shape apoptotic bodies [138]. The major signaling pathway regulating muscle mass and protein synthesis is the insulin-like growth factor 1 (IGF1)–AKT–MTOR pathway [139], which, when activated, increases muscle mass. Skeletal muscle also secretes myokines, which can increase insulin sensitivity, improve glucose handling, and regulate glucose and lipid metabolism, which affects energy metabolism and inflammation [140]. Myokines also regulate myogenic differentiation, fiber type switching, and muscle mass maintenance. Both muscle satellite cells and non-satellite cells are regulated by pro-inflammatory signaling and underlie muscle dysfunction, e.g., cachexia. The mitochondrial function of satellite cells is severely inhibited by pro-inflammatory signaling during cachexia, resulting in impaired myogenic processes and reduced muscle differentiation [141]. Other non-satellite muscle progenitors and accessory cells, including fibroblasts, fibroblast precursors (FAPs), and vessel-associated pericytes, produce an extracellular matrix (ECM) suitable for satellite cell renewal and muscle differentiation that is responsive to and regulated by cytokine and immune cells [142].

Mitochondria also play an important role in inflammation [143]. Mitochondria influence innate and adaptive immune activation through regulation of RNA sensor RIG-I (RIGI)/mitochondrial antiviral signaling protein (MAVS), NLR family pyrin domain containing 3 (NLRP3), and toll-like receptor 9 (TLR9) signaling pathways, as well as activated T cells (such as T regulatory cells) [144]. However, detailed knowledge of the innate and adaptive immunity involved in muscle inflammation is still lacking. Many musculoskeletal disorders, including cachexia, manifest systemic and local inflammation. The aggravated dysfunction and high lethality of cachexia are associated with systemic inflammation or activation of immune cells. An increase in pro-inflammatory signaling can reduce muscle-specific satellite cell differentiation. Among many other molecules involved, immune responses by damage-associated molecular patterns (DAMPs) and pathogen-associated molecular patterns (PAMPs) are the best-characterized cascades that trigger the inflammasome assembly and activate caspase-1 [145]. Caspase-1 activation leads to mitochondrial damage [146]. NFκB is an important signaling molecule, as well as the effector of chronic inflammation. NFκB activates MURF-1, which stimulates the proteasome machinery, thereby promoting the degradation of muscle cell proteins during muscle atrophy [147]. In addition, the pro-inflammatory cytokines interleukin 6 (IL6), TNF alpha (TNFα), transforming growth factor-beta (TGFβ), interferon gamma (IFNγ), and interleukin 10 (IL 10) have been shown to be important factors in muscle wasting [148,149].

## 4. Diagnostic Studies of Mitochondrial Damage and Clinical Treatment

### 4.1. Diagnosis of Mitochondrial Dysfunction in Skeletal Muscle

Diagnostic studies of mitochondrial damage have included blood and urine testing, exercise testing, histological and immunohistochemical analyses [150], enzymatic analysis of the OXPHOS complex [150], and genetic analysis of mtDNA (Table 1). In addition, whole-genome and whole-exome screening can be used to diagnose potential genetic abnormalities and disease-causing genes.

#### 4.1.1. Muscle Biopsy for Mitochondrial Dysfunction

Muscle biopsies can be used to diagnose compromised skeletal muscle due to mitochondrial disease [151]. For example, cryosections stained with modified Gomori’s trichrome show the presence of ragged red fibers (RRF) in skeletal muscle [152]. Features of the affected muscle include fractured, damaged mitochondria as well as abnormally enlarged mitochondria with a crystalline structure composed of globular inclusions and lipids [153,154]. Longitudinal muscle section studies have shown that RRF is a segmental abnormality involving a subset of muscle fibers, and these abnormalities are associated with OXPHOS, showing a respiratory chain dysfunction leading to abnormal mitochondrial proliferation [155].

#### 4.1.2. Molecular Genetics for the Identification of Mitochondrial Dysfunction

The deletion of mtDNA fragments is known to cause mitochondrial dysfunction with different clinical manifestations. Next-generation sequencing (NGS) can be used to detect mtDNA heterogeneity, point mutations [156], and mtDNA fragment deletion breakpoints [157]. Moreover, real-time PCR can be used to determine changes in the estimated mtDNA:nDNA ratio (mtDNA copy number) in the muscle tissue of patients with mitochondrial myopathy and provide diagnostic and prognostic assessments [158].

#### 4.1.3. Analysis and Measurement of Mitochondrial Respiration

Spectrophotometric assessment of individual OXPHOS activity is a method for the biochemical investigation and diagnosis of mitochondrial damage. With the use of fresh or frozen muscle homogenates, each complex is analyzed separately after oxidation/reduction of a specific substrate or substrate analog. For example, NADH dehydrogenase of complex I, succinate dehydrogenase of complex II, ubiquinone CYTC oxidoreductase of complex III, CYTC oxidase of complex IV, and ATP synthase of complex V (oligomycin-sensitive ATP synthase) (Figure 1). Additionally, fresh or frozen muscle tissue sections were analyzed separately after the addition of specific substrates or substrate analogs for oxidation/reduction. For example, COX/SDH histochemical testing showed CYTC oxidase (COX, complex IV)-negative fibers as blue, while normal COX-positive fibers were brown [159,160].

Differences in the expression of OXPHOS complex proteins can be determined by Western blot analysis or tissue immunostaining. For example, succinate dehydrogenase (SDH, complex II) histochemical analysis showed mitochondrial aggregation in the subsarcolemmal region of fibers after mitochondrial OXPHOS dysfunction led to mitochondrial proliferation. Blue native acrylamide–polyacrylamide gel electrophoresis (BN–PAGE) is an electrophoretic separation performed in the absence of denaturants, therefore, preserving interactions between individual subunits throughout the complex [161]. Individual OXPHOS complexes can be analyzed by activity staining using BN–PAGE bound gels, or they can be transferred to nitrocellulose membranes for immunoblotting to demonstrate the misassembly or deletion of complex structures [162].

Most intracellular ATP is produced by mitochondrial respiration. The cellular mitochondrial stress test is a method used to measure the key parameters of mitochondrial respiration. The Agilent Seahorse Extracellular Flux (XF; Agilent Technologies, Santa Clara, CA, USA) analyzer can simultaneously measure changes in pH and oxygen concentration around cells in culture medium over time, providing information on mitochondrial bioenergetic properties and functional status. The oxygen consumption rate (OCR) of cells is primarily related to mitochondrial respiration and is due to oxidase activity in the cytoplasm [163]. A baseline surrogate for glycolytic activity is the extracellular acidification rate (ECAR), reflecting intermediate pH readings for lactate and bicarbonate accumulation. The ECAR value is influenced by carbon dioxide released in the Krebs cycle that combines with water to produce carbonic acid [163].

**Table 1:** Diagnostic studies of mitochondrial damage.

Methods	Example or Directions
Blood and urine testing [164]	Serum lactate, CK, fasting glucose, TSH, ACP, OAU, etc.
Exercise testing [165]	Mitochondrial myopathy shows impaired or inefficient oxygen utilization with increased respiratory exchange
Electromyography [166]	Most clinical myopathies (such as inflammatory myopathies) have diagnostic abnormalities
Muscle histopathology [151,152]	Presence of multiple ragged red fibers with modified Gomori trichrome stain indicates compensatory proliferation of mitochondrial dysfunction
Electron microscopy [153]	Abnormal mitochondria with increased size and abnormal cristae
Muscle respiratory chain enzymology [159,160,161,162,163]	Frozen skeletal muscle tissue for respiratory chain enzymatic mutation
Molecular analysis [156,157,158]	Analysis of nDNA or mtDNA deletions, mutations, or copy number differences
ACP, acylcarnitine profiles; CK, creatine kinase; OAU, urine organic acids; TSH, thyroid-stimulating hormone.	

### 4.2. Clinical Treatment of Skeletal Muscle Disorders

The prognosis of patients with skeletal muscle disease caused by mitochondrial dysfunction varies widely depending on the type of disease and extent of organ involvement. Skeletal muscle disorders are accompanied by damage to multiple organs, leading to progressive weakness and possibly death. Although there is currently no specific treatment, maintaining efficient mitochondrial function may be beneficial. For example, aerobic and resistance exercise can increase the number and quality of mitochondria to maintain mitochondrial function or slow down deterioration, which can effectively reduce the rate of muscle atrophy caused by cachexia [116,124,167,168,169,170,171]. Exercise improves mitochondrial homeostasis in skeletal muscle disease by increasing gene expression of the mitochondrial fusion protein MFN2 and inhibiting mitophagy-related genes, as well as increasing SDH activity and ATP content [116,124]. In addition, vitamin therapy, such as the use of riboflavin and coenzyme Q, may subjectively improve fatigue and energy levels in some patients.

Mitochondrial transplantation, a possible therapeutic approach that involves injecting healthy mitochondria into damaged organs [172], has been used successfully in human pediatric patients with myocardial ischemia [173]. However, the mechanism of treatment remains unclear, and the efficacy of mitochondrial transplantation has been questioned [174]. However, it has been reported that mitochondrial transplantation can effectively treat various cell types and diseases, such as those involving cardiac and skeletal muscle, lung and liver tissues and cells, and neuronal tissues [175]. Therefore, mitochondrial transplantation may be one of the ways to treat mitochondrial dysfunction and delay the deterioration that occurs in affected patients. However, there are still discrepancies in the literature, and more work is needed to clarify whether mitochondrial therapy is beneficial for patients with skeletal muscle disorders.

## 5. Conclusions

Mitochondria are an important source of energy for maintaining normal skeletal muscle function. When mitochondria are defective or abnormal and cannot be effectively cleared or degraded, muscle disease or multisystem disease can result. Mitochondrial dysfunction may be caused by mitochondrial OXPHOS dysregulation, mtDNA mutations or deletions, imbalances in Ca^2^^+^ dynamics, altered expression or function of mitochondrial-related proteins, and ultrastructural defects. However, when coupled with an imbalance between mitochondrial fusion and fission, lysosomal dysfunction, and/or defective mitophagy, damaged mitochondria cannot be effectively cleared or degraded. Therefore, some questions remain: (i) What mechanisms are involved in mitochondrial repair and transplantation? (ii) What is the benefit and duration of skeletal muscle function recovery after mitochondrial repair? (iii) Are mitochondrial chaperones therapeutic targets or just biomarkers of certain skeletal muscle disorders? Further studies are needed to better understand how to effectively treat mitochondrial dysfunction-induced skeletal muscle disease.

## Data Availability

Not applicable.
